# Role of Clotrimazole in Prevention of Recurrent Otomycosis

**DOI:** 10.1155/2019/5269535

**Published:** 2019-12-26

**Authors:** Keyvan Kiakojuri, Ramazan Rajabnia, Saeid Mahdavi Omran, Abazar Pournajaf, Mohsen Karami, Mojtaba Taghizadeh Armaki

**Affiliations:** ^1^Department of Ear, Nose, and Throat, School of Medicine, Roohani Hospital, Babol University of Medical Sciences, Babol, Iran; ^2^Infectious Diseases and Tropical Medicine Research Center, Health Research Center, Babol University of Medical Sciences, Babol, Iran; ^3^Department of Microbiology, School of Medicine, Babol University of Medical Sciences, Babol, Iran; ^4^Department of Medical Parasitology and Mycology, School of Medicine, Babol University of Medical Sciences, Babol, Iran

## Abstract

Otomycosis is one of the relatively common diseases in the world which is caused by different fungi especially saprophytes. Concerning the relapse of this disease in a number of individuals, the present study was performed to evaluate the inhibitory effect of clotrimazole drop in the relapse of otomycosis. Clinical samples were taken by an ENT specialist from patients suspicious of having otomycosis. A part of these samples were stained, and others were cultured. The diagnosis of otomycosis was made on the basis of the recognizable and characteristic appearance of fungal hyphae or mycelium and fruiting bodies and/or conidiophores under microscopic examination. Patients with suspected otomycosis are not at risk of recurrence after treatment with clotrimazole drops. Out of the 161 individuals in whom definite diagnosis of otomycosis was made, the most affected individuals were, in the age range of 40–49 years, women, urban citizens, and housewives. Pruritus and diminished hearing were the main complaints of the patients. *Aspergillus niger* and *A*. *flavus* as well as *Candida albicans* were the main causes of the disease. The relapse of disease was observed in only five patients (3.1%), where *A. niger* was the main fungus. Most relapses were observed in women and in those with diminished hearing, manipulating the ears, ulcers in the canal, and tympanum. Our results suggested that usage of clotrimazole can be effective in reducing the relapse of otomycosis, and concerning the high cost of treating otomycosis while the low cost of using clotrimazole, usage of this drop is recommended to reduce the relapse of otomycosis.

## 1. Introduction

Otomycosis is one of the relatively common diseases in the world including Iran, claiming around 30% of ear infections [1–7]. Among the contributing and stimulating factors for development of otomycosis are manipulating the ears, humidity, heat, age, predisposing primary bacterial infection, and immune system disorders [8–12]. The main clinical findings of otomycosis include pruritus, scaling, discharge, and pain [9, 13]. This disease involves individuals in different ages ranging from infanthood to advanced age (81 years) with the mean age of 30–40 years. There is no significant difference between men and women or urban and rural citizens [4, 6, 14–17]. The direct examination of diagnosis of ear fungal infection is made by observing fungal elements including mycelium, pseudomycelium and yeasts in the samples of the discharge, cerumen, and scales of the ear canal [18].Several fungi cause otomycosis, of which the most common are saprophytes (70%), including *Aspergillus* spp. and *Fusarium* spp., yeasts (20 -25%), and dermatophytes (about 5%) [15, 19]. The main causative agent of otomycosis among saprophytes and yeasts are *Aspergillus niger* and C. *albicans*, respectively [6, 10, 13, 20, 21]. The other critical species including *A. flavus* and *A. fumigatus* are gradually finding a progressive role in development of otomycosis [3, 4, 19]. The other causative agents are *Cladosporium* spp. [12], *Alternaria* spp. [21], *Mucor* spp., and *Rhizopus* spp. [21, 22]. The minimum role is played by dermatophytes which have been reported in a very few cases [5, 21, 23].

In some investigations, only *A. niger* and *C. albicans* were the cause of otomycosis [24]. In some cases, the fungi were observed only in the masses present in the ear canal (cerumen) without any sign of disease in the ear [14, 21, 25, 26]. Several treatment procedures are used according to the need of the patient with otomycosis. In the treatment of otomycosis, usually first fungal elements are removed from the ear (by suction or washing) and then dried. The medications used given the type of fungus include clotrimazole or miconazole, which are used in combination with antibacterial drugs such as ceftazidime [12, 27, 28]. Nevertheless, some compounds with disinfection properties such as betadine and boric acid in combination with miconazole have also been used [12, 24]. In practice, the physicians' recommendations usually improve the complaints within 1-2 weeks without any side effects [20, 27]. In contrast, several reports are available about otomycosis treatment failure varying between 5.88 and 17% [10, 29, 30]. The relapse of otomycosis occurred in some patients, due to different reasons including improper selection or prescription of drugs, not performing complementary treatments, or incomplete consumption of drugs. The rate of relapse varied from 7 to 48% [6, 17, 30]. In this regard, in a comparative study, Berjis et al. observed that 36.4% of those who used clotrimazole for the treatment experienced relapse and 33.3% of patients who had used tolnaftate for treating otomycosis had also experienced relapse of otomycosis [27]. Since, in our previous study, it was observed that otomycosis relapse existed in 7.3% of cases [31], the present study was performed with the aim of determining and reducing the relapse among cases having otomycosis during 2017-2018 using clotrimazole for 1 month after primary treatment of the disease in Mazandaran Province, Babol, Iran.

## 2. Materials and Methods

The individual who referred to ENT ward of Ayatollah Rouhani Hospital in Babol for problems in their ears were evaluated by the physician. The patients were included if they are suspicious of having otomycosis including no rupture of the tympanic membrane, existence of scaling, pruritus, dark and white discharge, as well as cotton-like masses in microscopy investigation of the ears. Their primary information including age, gender, duration of having disease, place of residence, and job were obtained by using the questionnaire form. Then, the complaints of the patient including discharge, scaling, pruritus, pain, hearing loss and the physician observation in the canal and tympanic membrane were recorded. Discharge, scaling, or ear mass was taken by an ENT through sterilized instruments. Then, a spread of it was prepared on slide and stained using Gram method. A part of these samples was cultured on Sabouraud dextrose agar supplemented with chloramphenicol (Sc) and/or Sc medium supplemented with cycloheximide (Scc) for possible observation of fungal colonies, and kept at 25°C and 37°C at most for at least four weeks. In addition, conventional measures were also performed for diagnosing the possible presence of bacteria in the ears, including culture on blood agar, chocolate agar, and differential tests. The diagnosis of otomycosis was made by observing fungal elements in direct examination that include mycelium or pseudomycelia, yeast, or arthroconidia. The fungi were detected based on macroscopic and microscopic morphology as well as by using other conventional methods such as slide culture and germ tubes. In case of not observing fungal elements or bacterial growth, or negativity of both, the subject of interest was excluded from the current study. For all of the individuals, common therapeutic methods were performed. They included clearing and drying the canal and use of systemic and topical drugs (ointment and drops) for 4 weeks. After this duration, the patients were referred to ENT again for investigating the ear canal. At this stage, the ear canal was controlled in terms of elimination or improvement of symptoms. If the therapeutic outcomes were satisfactory, the patient was transitioned to the preventive treatment stage. At this stage, the patient received clotrimazole drops (three times in week, each time two drops) in the ear, after which the relapse or complete elimination of the disease was evaluated.

### 2.1. Statistical Methods

The obtained information was sorted in SPSS software version 22, where the qualitative data were expressed as percentage and ratio, while the quantitative data were expressed as mean and standard deviation. Chi-do, Fisher-exact, and *T*-test were used for analyzing quantitative and qualitative data. The significance level for all methods was considered as *p* ≤ 0.05.

## 3. Results

Our results indicated that of the 207 subjects who underwent primary assessment and were suspicious of having otomycosis, 161 (77.78%) of them had positive direct test result and in turn positive fungal culture. Due to the absence of fungal elements in microscopic examination and bacterial contamination in culture media, 46 samples were excluded. The age range of 161 individuals with otomycosis was 1–86 years, whose mean age was 43.11 ± 19.25. Of them, the majority of the affected individuals (20.5%) were 40 −49 (n = 33) years, while the minimum number of individuals (2.5%) were within the age range of 1–9 years (*n* = 4). Nevertheless, no significant difference was observed between the age groups in the Chi-do test (Table 1). The majority of the individuals with otomycosis (43.5%) were housewives (*n* = 70), followed by those with freelancing jobs and students (*n* = 37, 23%; *n* = 25, 15.5%) (Table 2). The place of residence of most affected individuals 133 (76.4%) was various urban regions in Mazandaran province. Women (58.4%; *n* = 94) were more affected than men (41.6%) by otomycosis. Among the most important predisposing factors were use of cotton swab, matches, and fingers, with 58 (36%), 29 (18%), and 4 (2.5%), respectively. The results of investigation showed that 84 cases (52.2%) of affected individuals had an infection in the left ear. None of the patients had otomycosis in both years. Pruritus, diminished hearing, and discharge, with 87%, 83.2%, and 69.6%, respectively, were among the most common clinical signs in individuals with otomycosis (Table 3). The course of disease in the subjects varied between 1 and 180 months, whose mean was 25.17 ± 14.81. Investigation of ENT on the canal and tympanum indicated that in 39.8% of the affected individuals (*n* = 64), the surface of the canal and tympanum had ulcers and scars. In the present investigation, limited fungi were involved in the development of infection in the ears of these subjects. The most common fungi were from different species of *Aspergillus* spp. with 129 cases (80%) as well as different species of *Candida* with 31 cases (19.3%). The most important species of *Aspergillus* isolated from the individuals with otomycosis with 61 cases (37.9%) was *A. niger*; *C. albicans* with 23 cases (14.29%) was another important species of *Candida* (Table 4). Disease relapse was observed in only five patients (3.1%); recurrence was seen only in 4 women, 3 villagers, and 3 people who cleaned cerumen with cotton swabs. The job of three of them was housewife and the rest were freelancer. Four of them had diminished hearing. The age of individuals with otomycosis relapse with 47.6 ± 10.5 years was higher than the mean total age of the affected subjects. However, the course of disease in these individuals was shorter (13.4 ± 12.01 months). The statistical methods used in this regard showed no significant difference. Further, 80% of relapse cases were observed in those who had ulcers in the canal or tympanum. In three of them, the fungus observed in both stages was *A. niger*; in both cases, due to few number of samples, even with Fisher's statistical test, no significant difference was observed between ulcer or type of fungus and relapse.

## 4. Discussion

The present study was performed with the aim of determining the role of clotrimazole in preventing the relapse of otomycosis in Babol. Here, of the 207 individuals with external ear infection, 161 (77.78%) had otomycosis. This result is in line with the findings of some investigations which showed that the frequency of otomycosis among the studied individuals was high (57–78%) [4, 32, 33]. This high rate of otomycosis first indicates greater precision in such research and highlights the progressive importance of fungi in development of ear infections due to different reasons including climate status. Nevertheless, the role of extensive use of antibacterial antibiotics, immune system compromising drugs, and steroids should also be taken into account [34]. In some reports, the frequency of otomycosis is reported as low (22.8–38%) but higher than in the official books and references which report the role of fungi only as 10% [16, 35]. Nevertheless, the difference in the prevalence of otomycosis across different investigations can be due to the inclusion criteria, not performing proper clinical and laboratory diagnostic methods for diagnosis of fungi, not taking some fungi into account which are rarely involved in otomycosis, and the geographical location studied [36]. Presence of specific clinical signs and symptoms in the ear of individuals with ear infection plays a significant role in ENT's suspicion to otomycosis. In the present study, pruritus, diminished hearing, and discharge have been the major clinical symptoms in those with otomycosis, which is in line with the results of most investigations in this regard [1, 6, 10]. Nevertheless, typically, these symptoms are also observed with differences in bacterial infections of the ear [15]. The disease of all those with otomycosis in the present study had clinically improved completely during two weeks of treatment, and no sign of disease was observed in ear re-examination. However, in some investigations, treatment failure (9–17%) has been reported. This failure can be due to the type of drug chosen, disregarding the possibility of resistance of the organism causing this disease, not completing the therapeutic course [27, 37, 38], genetic variations, surgery, or use of hearing assistive technology systems [10, 11, 29, 30]. As an important finding of the present study, of the 161 individuals with otomycosis, only 3.1% of them had relapse after completing the therapeutic course and stated complete improvement of the disease by ENT and the patient satisfaction with their treatment. Various studies have suggested that the relapse of otomycosis can be different. Only in one study, no observation of relapse has been reported [20]. Nevertheless, other reports in which no relapse has been mentioned can also be added though they have not been cited here because of unreliability. In another investigation, otomycosis relapse was reported as 48% [30], and another study reported 14.29% [39]. Apart from the few mentioned investigations, in most studies, otomycosis relapse has been reported as less than 9% (7.1–8.8%) [6, 17, 28, 40]. Overall, comparison of these results suggests the reduction of the rate of relapse of otomycosis due to using clotrimazole drop in the present study. It should be noted that in other reports, use of a disease-preventing drug has not been mentioned. Nevertheless, application of different drugs for reducing the development or relapse of dsisease and decreasing the referral of patients including not using antibiotics [4] and miconazole ointment [41] had been proposed. In the present study, in order to prevent relapse of otomycosis, clotrimazole drop was used for one month following the primary treatment in these patients. Therefore, achieving such a result can be logical. The causative fungi of otomycosis in the present study are similar to those reported some other research [6, 8, 10]. Nevertheless, in spite of the relative similarity in terms of fungal causes in this investigation and other studies, the obtained results are different [20, 30, 39]. This can be due to underlying diseases such as diabetes, manipulating the ears, and previous surgical operations [9, 10, 16, 30]; as in the present study, 80% of those with relapse had some history of ear manipulation. Nevertheless, the age of patients due to different immunity of the subjects can also be responsible these differences. In this regard, in some investigations, the mean age of the individuals was higher [17, 20, 30] or lower [6, 10] than that in the present study. Also the gender of patients can also affect this disease, since in the present study, most of those who had otomycosis and its relapse were female, where the relapse was observed mostly in housewives, which are somehow similar to other studies [8, 10]. However, in some studies, men more affected than women and no better therapeutic response had been observed [6, 20, 30, 39]. If the number of studied patients does not reach an acceptable level, its results cannot be compared with other studies [6, 17]. However, if the number of studied individuals increases, the rate of relapse would become more realistic [20]. Nevertheless, in some cases, its opposite has also been observed [30]. So, other factors such as socioeconomic conditions [20] or compromised immune system [36] should also be taken into account. Paz Cota et al. [42] confirmed the effectiveness of eberconazole 1% at improving clinical symptoms of otomycosis and resolving fungal infection. In Mofatteh et al.'s study [24], the efficacy of clotrimazole and betadine solution 10% treatments was equal in management of otomycosis. Dundar and İynen [43] conducted a prospective study on 40 patients with otomycosis. In this study, the ear canal was filled with 1% clotrimazole, using an intravenous catheter and syringe [43]. The authors described the efficacy of single clotrimazole 1% was good for the treatment of otomycosis [43]. Swain et al. [44] showed a study on 44 recalcitrant otomycosis patients who were divided into two groups. One group was treated with clotrimazole and the other group was treated with povidone iodine. The povidone iodine treatment at recalcitrant otomycosis patients was effective and well tolerated [44]. Omran et al. [45] showed that the use of combination treatment with ceftizoxime and clotrimazole drugs was useful in treatment of the otomycosis patients with tympanic membrane rupture. In Kiakojori et al.'s study [31], 2% miconazole ointment was an effective treatment in cases with otomycosis. The results of the present study showed that clotrimazole can have a preventive role in the relapse of otomycosis. Major cases of relapse were observed in those who had inflamed or ulcerated canal and tympanum. Based on the obtained result, it is suggested that in those with otomycosis, especially in individuals with disorders in their ears and tympanum, clotrimazole drop can be used to prevent the relapse of this disease. Concerning the economical price of this drug, it will result in decreased discomfort, therapeutic cost, and time waste of patients.

## Figures and Tables

**Table 1:** Age distribution in patients with otomycosis in Babol, north of Iran.

Age groups	Number	Percent
1–9	4	2.5
10–19	14	8.7
20–29	28	17.4
30–39	22	13.7
40–49	33	20.5
50–59	28	17.4
60–69	14	8.7
70–79	12	7.5
80–89	6	3.7
Total	161	100

**Table 2:** Job distribution in patients with otomycosis in Babol, north of Iran.

Job	Number	Percent
Unemployed	2	1.2
Student	25	15.5
Housekeeper	70	43.5
Employee	4	2.5
Self-employment	37	23
Farmer	14	8.7
Teacher	2	1.2
Retired	7	4.3
Total	161	100

**Table 3:** Sign and symptoms in patients with otomycosis in Babol, north of Iran.

Sign	Number	Percent
Itching	140	87
Hearing lose	134	83.2
Discharge	112	96.6
Inflammation	90	55.9
Pain	71	44.1

About 70% of patients referred to doctor with more than one complaint in ear.

**Table 4:** The etiological fungal agents in the otomycosis in the present study.

Fungus	Number	Percent
*A. niger*	61	37.89
*A. flavus*	53	32.92
*A. fumigatus*	6	3.73
*A. terreus*	9	5.59
*Fusarium* spp.	1	0.62
*C. albicans*	23	14.29
*C. tropicalis*	3	1.86
*C. krusei*	4	2.48
*Candida* spp.	1	0.62
Total	161	100

## Data Availability

The data used to support the findings of this study are available from the corresponding author upon request.
